# Polarization-driven twisted states in ferroelectric nematic liquid crystals under confinement

**DOI:** 10.1038/s41598-026-48218-7

**Published:** 2026-04-17

**Authors:** Anna Savchenko, Ebba Grönfors, Rachel Tuffin, Melanie Klasen-Memmer, Per Rudquist, Frank Giesselmann

**Affiliations:** 1 Institute of Physical Chemistry, University of Stuttgart, 70569 Stuttgart, Germany; 2 Department of Microtechnology and Nanoscience, Chalmers University of Technology, 41296 Gothenburg, Sweden; 3 Merck Electronics KGaA, 64293 Darmstadt, Germany

**Keywords:** Materials science, Physics

## Abstract

Ferroelectric nematic liquid crystals (FNLC) are 3D fluids with a giant spontaneous electric polarization ($\textbf{P}$) in the order of several microcoulombs per centimeter squared. In an unconstrained sample this high $\textbf{P}$ has recently been shown to twist the nematic director field in order to reduce the electrostatic energy [P. Kumari et al., Science 383, 1364 (2024)]. By studying an FNLC, namely AUUQU-2-N, in a wedge cell with continuously increasing thickness, we now show that the polarization-driven twist modes depend on the local distance *d* between the lower and the upper plates of the wedge. For planar and parallel anchoring conditions of the nematic director we find a uniform, non-twisted director field at small *d* below 2 $\mu \text {m}$ and a likely $2\pi$-twisted director field above a certain critical thickness of about 5 $\mu \text {m}$. At intermediate *d* we observe locally twisted director fields but with zero total twist between the lower and the upper surface. We coin these twisted director configurations with alternating twist sense ”mesotwisted”. In view of these polarization-driven twist instabilities in FNLCs, the uniform state at small *d* might be considered as a surface-stabilized ferroelectric nematic, an interesting analogy to surface stabilized ferroelectric chiral smectics.

## Introduction

**Fig. 1:**
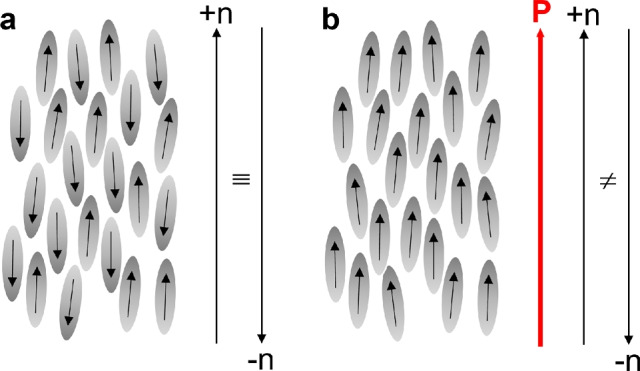
Schematic representation of nematic $\text {N}$ (**a**) and ferroelectric nematic ${\text {N}}_{\text {F}}$ (**b**) phases. Grey ellipsoids represent rod-shaped mesogenic molecules with longitudinal dipole moments indicated by the arrows.

In the recently discovered ferroelectric nematic (${\text {N}}_{\text {F}}$) liquid crystal phase, the polar ordering of rod-shaped molecules with large longitudinal dipole moments breaks the director sign invariance $+ \textbf{n}= - \textbf{n}$ of conventional (non-ferroelectric) nematics (N) and gives rise to giant spontaneous electric polarization ($\textbf{P}$), cf. Fig. 1^1–8^. The magnitude of this polarization is comparable to that of solid-state ferroelectrics, making electrostatic energy a significant contribution to the free energy and potentially reaching the order of elastic contributions^9^. For example, splay deformations, very common in non-ferroelectric nematics, are avoided in ${\text {N}}_{\text {F}}$ phases since the bound charge density $\rho = - \nabla \cdot \textbf{P}$ increases the electrostatic energy. Polarization-related charges are also avoided at interfaces such that $\textbf{P}$ aligns preferentially parallel to surfaces and domain walls.

**Fig. 2:**
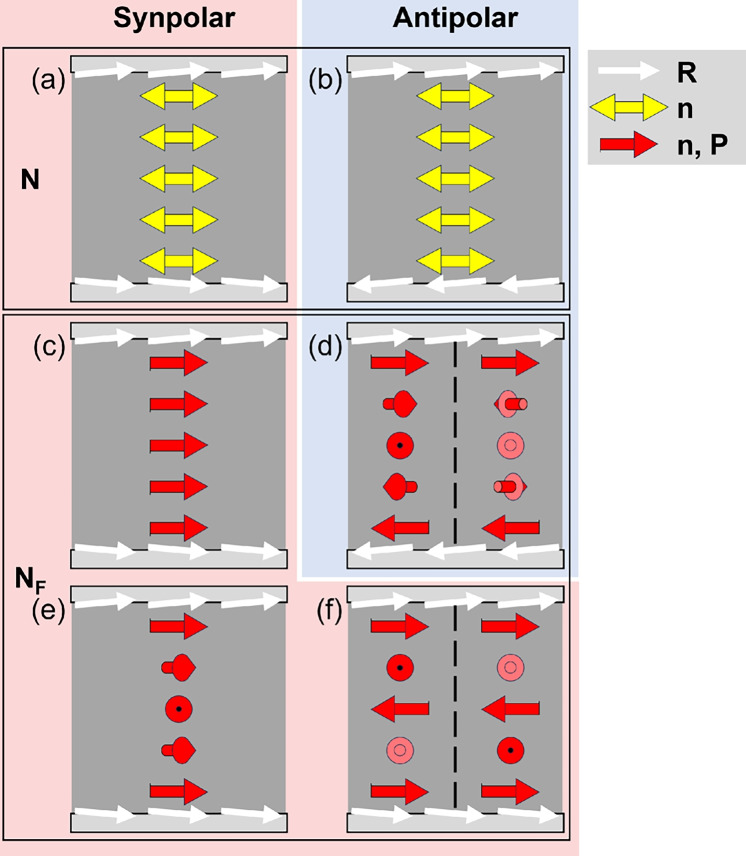
Planar director configurations of nematic (N) and ferroelectric nematic (${\text {N}}_{\text {F}}$) phases in synpolar cells (with parallel rubbing directions $\textbf{R}$ at the boundaries, red background) and in antipolar cells (with antiparallel $\textbf{R}$ at the boundaries, blue background). (**a**, **b**) Uniform axial alignment of the nematic director $\textbf{n}$ in both (**a**) synpolar and (**b**) antipolar cells. (**c** - **f**) Configurations of the ${\text {N}}_{\text {F}}$ director and polarization $\textbf{n,P}$ assuming that the polarization $\textbf{P}$ aligns parallel to $\textbf{R}$. (**c**) Uniform polar $\textbf{n,P}$-configuration in a synpolar cell^10^, (**d**) $\pi$-twisted $\textbf{n,P}$-configuration in an antipolar cell^10^, (**e**) mesotwisted $\textbf{n,P}$-configuration in a synpolar cell where the twist-sense reverses at the midplane such that the total twist across the cell remains zero and (**f**) $2 \pi$-twisted $\textbf{n,P}$-configuration in a synpolar cell. In this paper we will present experimental support for the configurations in (**e**) and (**f**).

It is well-known that rubbed polymer layers, e.g. certain polyimides, axially align conventional nematics with director $\mathbf {\pm n}$ parallel to the rubbing direction $\textbf{R}$, cf. Fig. 2a and b. In ferroelectric nematics a unidirectionally rubbed surface polarly aligns $\textbf{P}$ either parallel or antiparallel to the rubbing direction^10–12^. For reasons of simplicity, let us now assume that close to a rubbed surface the direction of $\textbf{P}$ aligns parallel to the direction of rubbing $\textbf{R}$ of that surface. Under these polar in-plane anchoring conditions, the relative rubbing directions of the lower and upper glass substrates of a cell become crucial. Following the terminology in^10^, we call a cell with lower and upper surfaces rubbed in the same direction ”synpolar” and a cell with lower and upper surfaces rubbed in opposite directions ”antipolar”. The parallel rubbing directions in a synpolar cell allow for a uniform (non-twisted) arrangement of $\textbf{n}$ and $\textbf{P}$ as shown in Fig. 2c^10^. In an antipolar cell however, the antiparallel rubbing directions force $\textbf{P}$ and $\textbf{n}$ to twist $\pm 180^\circ$ between the surfaces to match the polar in-plane anchoring conditions as seen in Fig. 2d. Since in the absence of any external forces the twist might be either right- or left-handed, twist domains with opposite handedness appear^10^.

So far, the presence or absence of a twist has been thought to solely depend on the boundary conditions imposed by the surfaces. However, this picture seems too simplistic, as it neglects the impact of electrostatic energy arising from the large $\textbf{P}$ in the ${\text {N}}_{\text {F}}$ phase. While a solid-state ferroelectric minimizes its electrostatic energy by breaking up into domains with opposite directions of $\textbf{P}$^13^, a fluid ferroelectric, such as ${\text {N}}_{\text {F}}$, has the possibility to continuously twist the direction of $\textbf{P}$ in space, as was pointed out by Khachaturyan fifty years ago^14^. In fact, such a polarization-driven twist was recently observed by Kumari et al.^15^ in unconstrained samples of the archetype ${\text {N}}_{\text {F}}$ materials DIO^2^ and RM734^1^. Signs of it have also been seen in constrained samples of DIO^16^ and of RM734^17^. The $\textbf{P}$-driven twisting of the ${\text {N}}_{\text {F}}$ director along a certain direction $\textbf{z} \perp \textbf{n}$ is counteracted by twist elasticity. The balance between electrostatic and twist elastic energy contributions defines a kind of ”preferred twist”. As long as the molecules are non-chiral, the $\textbf{P}$-driven twist can be left- or right-handed with the same probability.

We show that this $\textbf{P}$-driven twisting also seems to occur in constrained samples. In particular, in the ${\text {N}}_{\text {F}}$ material AUUQU-2-N it leads to twisted structures even in synpolar cells, depending on their thickness. In synpolar wedge cells we find three regimes: a uniform, non-twisted director field at small *d* below 2 $\mu \text {m}$ and a twisted director field above a certain critical thickness of about 5 $\mu \text {m}$. As the polar anchoring at rubbed surfaces has been indicated to be strong^10^ it is reasonable to assume that it is a $2\pi$ twist (Fig. 2f), an assumption which is also supported by a tendency to form $2\pi$ twists presented by Paik and Selinger^18^. At intermediate *d* we suggest that there is a locally twisted director field but with zero total twist (”mesotwist”) as shown in Fig. 2e. It has not escaped our notice that one important implication of our findings is that the uniform state at small *d* might be considered as a surface-stabilized ferroelectric nematic, an interesting analogy to surface stabilized ferroelectric chiral smectics^19^.

## Results

**Fig. 3:**
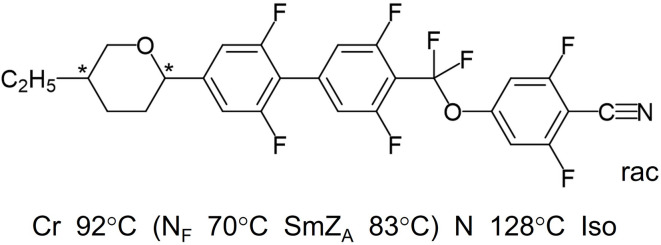
Chemical structure and phase sequence of the FNLC mesogen AUUQU-2-N. The racemic mixture was used in all experiments.

In the study we used AUUQU-2-N (Merck Electronics KGaA, 64293 Darmstadt, Germany)^20^, with the molecular structure and phase sequence depicted in Fig. 3. The monotropic Sm${\text {Z}}_{\text {A}}$ and ${\text {N}}_{\text {F}}$ phases are metastable and appear only in cooling from the isotropic phase. The in-plane polar surface alignment of FNLCs on a polyimide surface rubbed along $\textbf{R}$ is parallel to $\textbf{R}$, either $\textbf{P}\!\uparrow \uparrow \!\textbf{R}$ or $\textbf{P}\!\downarrow \uparrow \!\textbf{R}$ depending on the mesogen and polyimide. For our analysis it does not matter if it is $+\textbf{P}$ or $-\textbf{P}$ that aligns with $\textbf{R}$ in the case of AUUQU-2-N. For simplicity we have assumed that $\textbf{n, P}\!\uparrow \uparrow \!\textbf{R}$.

**Fig. 4:**
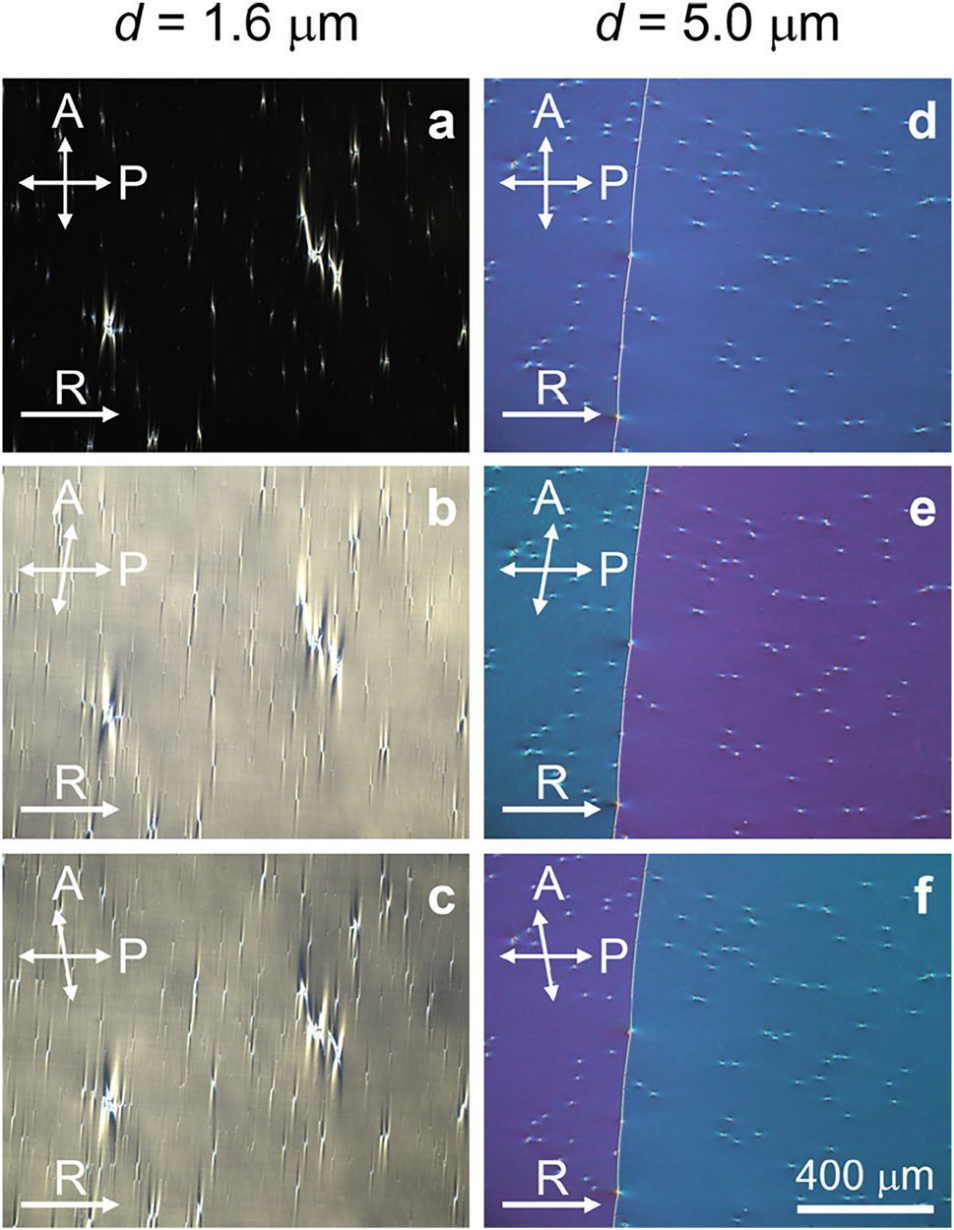
Optical textures of the ${\text {N}}_{\text {F}}$ phase of AUUQU-2-N in synpolar cells using polarized optical microscopy. The material was filled in $1.6~\mu \text {m}$ (**a**-**c**) and $5.0~\mu \text {m}$ (**d**-**f**) cells with planar synpolar anchoring conditions. The parallel rubbing directions of both bottom and top substrates (synpolar) are horizontal in (**a**-**f**). Texture micrographs were recorded at $65^{\circ }\textrm{C}$ with crossed and $\pm 10^\circ$ decrossed polarizers.

Polarized optical microscopy (POM) studies of the ${\text {N}}_{\text {F}}$ phase in $1.6~\mu \text {m}$ and $5.0~\mu \text {m}$ thick synpolar cells revealed different director structures at different cell thicknesses. For a thin synpolar cell, $d = 1.6~\mu \text {m}$, a uniform ${\text {N}}_{\text {F}}$ state is obtained, Fig. 4 a-c. Under crossed polarizers the cell appears dark when the director is parallel to one of the polarizers, except near the conic defects^21^. The defects are characteristic for the phase and occur at the glass spacers as well as at impurities in the cell. In the $d = 1.6~\mu \text {m}$ cell, the conic defects run more or less normal to the rubbing direction $\textbf{R}$. The uniform planar alignment corresponds to the schematic representation given by Fig. 2d.

The situation in a $d = 5.0~\mu \text {m}$ cell is very different. On cooling from the isotropic phase through N and Sm${\text {Z}}_{\text {A}}$ to $\text {N}_\text {F}$, below $70^\circ$ C a uniform texture with conic defects appears first. At around $\sim 1^\circ$C below the transition, we observe structural changes resulting in the formation of twist domain walls. The domain walls separate domains with oppositely twisted states (see Fig. 4d) revealed by de-crossing of the polarizers. When the polarizers are decrossed clockwise, the twisted states appear with different colors (Fig. 4e), which are interchanged when the polarizers are decrossed the same angle but counterclockwise (Fig. 4f). This is characteristic of twisted domains having the same magnitude, but opposite handedness of twist. Interestingly, now the conic defects run parallel to $\textbf{R}$ and the domain walls are normal to $\textbf{R}$. These changes are observed during the phase transition from Sm${\text {Z}}_{\text {A}}$ to $\text {N}_\text {F}$, see Supplementary Information.

A wedge cell with a cell gap continuously increasing from $d \approx 0.8\,\mu \text {m}$ to $d \approx 9.2\,\mu \text {m}$ was fabricated in order to study the evolution of director configurations with increasing *d*. The glass plates were rubbed unidirectionally (synpolar arrangement) with the rubbing direction oriented normal to the thickness gradient. The filled wedge cell was slowly cooled from the isotropic phase at a rate of 0.5 K/min even though higher rates (up to 5 K/min) appear to have no significant impact on the results.

**Fig. 5:**
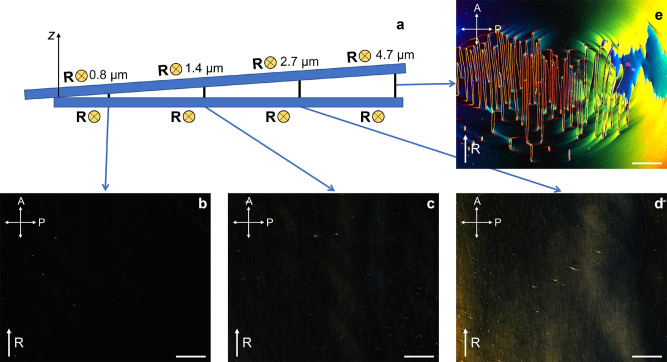
(**a**) Schematic of the synpolar wedge cell with rubbing direction R (indicated by the yellow vectors pointing into the paper plane) normal to the gradient of cell thickness. Texture micrographs of the ${\text {N}}_{\text {F}}$ phase taken between crossed polarizers in the synpolar wedge cell at (**b**) cell gap of $0.8~\mu \text {m}$, (**c**) $1.4~\mu \text {m}$, (**d**) $2.7~\mu \text {m}$, and (**e**) $4.7~\mu \text {m}$. The cell gap is given for the middle of each photo. At low cell thicknesses extinction of light is observed between crossed polarizers (**b**,**c**). At medium cell thicknesses around $2.7~\mu \text {m}$ no extinction is observed regardless of cell rotation between the crossed polarizers (**d**). At higher cell thicknesses around $d = 4.7~\mu \text {m}$, disclinations appear and twisted domains are formed (**e**). The scale bars in (**b**)-(**e**) are each $200~\mu \text {m}$.

POM studies show that the director field is homogeneous in the $\text {N}$ and Sm${\text {Z}}_{\text {A}}$ phases in the whole wedge cell. Full extinction is obtained when $\textbf{R}$ is along one of the crossed polarizers. After phase transition to ${\text {N}}_{\text {F}}$, however, the texture remains homogeneous only in the thinnest part of the cell (Fig. 5b,c). At the cell gaps of $0.8\,\mu \text {m}$ and $1.4\,\mu \text {m}$ light extinction is preserved except in areas around conic domains. At $d = 2.7~\mu \text {m}$, light passes through the non-decrossed analyzer (Fig. 5d). Extinction of light can not be obtained for any azimuthal position of the cell between crossed polarizers, indicating a non-uniform alignment of the director along the *z*-axis. At cell thickness of $4.7\,\mu \text {m}$ twist domains are formed (Fig. 5e), similar to the ones shown for the sandwich cell in Figures 4d-f. These findings indicate the existence of a *transition state* between the uniform and twisted director states. This transition state is not uniform but has to be topologically equivalent to a uniform state since they can exist in contact with each other under the same surface alignment conditions without any discontinuity between them.

An interesting difference between the situations in the wedge cell and the flat 5 $\mu$m cell is that the domain walls run preferentially normal to the rubbing direction $\textbf{R}$ in Fig. 4d-e while they run preferentially parallel to $\textbf{R}$ in the wedge cell in Fig. 5e. The reason for this striking difference is still unclear to us. However, it might be related to additional constraints imposed by the spatial coexistence of twist domains and mesotwisted states in the same wedge cell. This spatial coexistence of director configurations with different topology is not found in a flat cell that is either uniform, mesotwisted, or twisted.

**Fig. 6:**
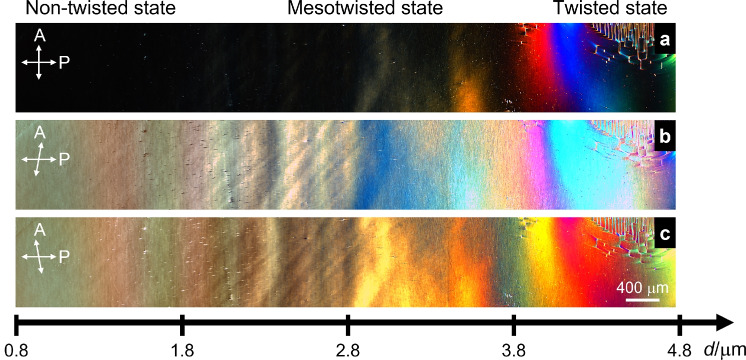
Panorama picture of a large part of the synpolar wedge cell filled with the ${\text {N}}_{\text {F}}$ phase under crossed (**a**) and $\pm 25^\circ$ decrossed polarizers (**b**,**c**). In the thinnest part (left) of the wedge cell there is an extinction point between crossed polarizers. Absence of optical divergence while decrossing polarizers indicates uniform director alignment in this part. At the thicker cell gap (right) optical activity and formation of disclinations is due to formation of twisted domains. In the intermediate area the optical divergence when uncrossing polarizers indicates an intermediate state between uniform director field and fully twisted states. The black scale indicates the gradually increasing thickness of the cell.

Figure 6 shows a picture of the thinner half of the wedge cell. Multiple photos were taken by shifting the cell while keeping the camera settings constant, and these were then joined together to form a panorama covering a large continuous part of the cell. The pictures were taken under crossed polarizers (Fig. 6a) and with decrossed polarizers $\gamma = 65^{\circ }$ (Fig. 6b) and $\gamma = 115^{\circ }$ (Fig. 6c), where $\gamma$ is the angle between the polarizer and the analyzer. The rubbing direction is parallel to the plane of polarization of the first, fixed, linear polarizer. In the thinnest part of the cell, $0.8 ~\mu \text {m}<d < 1.6 ~\mu \text {m}$, a uniform state is observed, supported by the existence of an azimuthal wedge orientation where extinction of light is achieved between crossed polarizers, with the same color being revealed upon both clockwise and counterclockwise de-crossing.

**Fig. 7:**
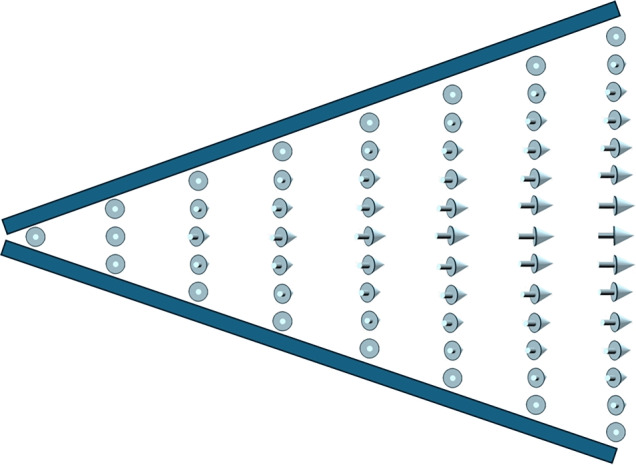
Schematic illustration of the appearance of a mesotwisted $\mathrm {N_F}$ director field in a wedge cell with synpolar boundary conditions. In the thin area of the cell (left), the director field is uniform. The longer the distance between the lower and upper boundaries, the more the director twists into the plane of the paper. At the horizontal midplane, the twist sense reverses such that the synpolar boundary conditions are fulfilled at both the upper and lower boundaries. Please note that the horizontal midplane is a mirror plane of the director field, meaning the entire structure is achiral, even though both the upper and lower halves of the cell are chirally twisted. This is similar to the achiral molecule of meso-tartaric acid, which has two chiral centers, which are mirror-symmetric to each other.

In the cell gap range of $1.6 \ \mu \text {m}< d < 4.0 \ \mu \text {m}$ the rotation of the analyzer in relation to the polarizer indicates a non-uniform state. This is evidenced even in instances where the cell appears black between crossed polarizers. It is observed that the colors revealed by de-crossing to $\gamma = 65^{\circ }$ differs significantly from those revealed at $\gamma = 115^{\circ }$. This observation indicates that the director structure is not symmetric with respect to $\textbf{R}$ and that there is no waveguiding effect (Mauguin limit). We propose this new state to be a pre-transition state, since, despite the director field not being uniform, there are no indications of formation of twist domain walls or disclinations. On the one hand, the optical asymmetry, namely the interchange of colors when decrossing the polarizers in opposite directions, suggests that this state is somehow chirally twisted. On the other hand, the absence of twist domains indicates that the total twist of this state is zero and that it is thus achiral overall. We show a schematic example of such a structure in Fig. 7 and suggest the name *mesotwist* for these locally twisted but globally achiral states. The name is inspired by the stereochemical term of ”meso compounds”, molecules that have two or more chiral centers but which are achiral overall since they can be superimposed with their mirror image.

For cell gaps $d> 4.7 ~\mu \text {m}$, the director of the phase develops twist domains. Depending on the direction of de-crossing the analyzer regions separated by the twist domain walls are getting brighter or darker with interchange of color. This finding suggests that the director structure exhibits regions with similar twists but opposite twist sense separated by the internal domain walls.

**Fig. 8:**
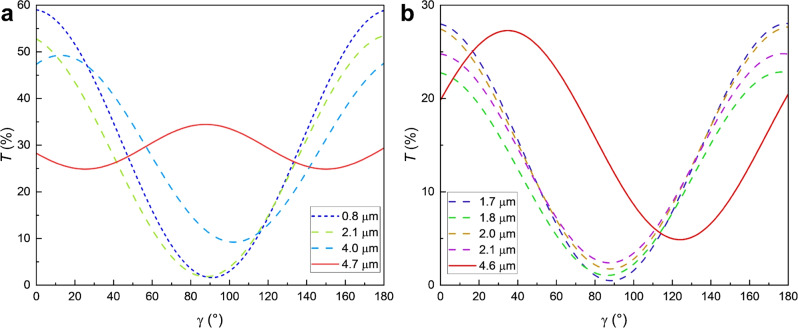
Transmitted light intensity *T* of a synpolar wedge cell filled with the ${\text {N}}_{\text {F}}$ phase versus the angle $\gamma$ between the analyzer and the polarizer, measured at room temperature and at different cell thicknesses for (**a**) white light with an optical center at 550 nm and (**b**) for monochromatic light with 589 nm wavelength. The rubbing direction is aligned along the polarizer.

Transmitted light intensity was measured at different cell gaps along the wedge as a function of the analyzer position $\gamma \in [0^\circ ,180^\circ ]$, with the fixed polarizer parallel to the rubbing direction, see Fig. 8. At $d = 0.8 ~\mu \text {m}$ and $\gamma = 90^{\circ }$, the light transmittance is $T \approx 0\%$. The transmittance increases with the dependency $T(\gamma ) \propto \sin ^2(\gamma -90^\circ )$. The data shows that the ${\text {N}}_{\text {F}}$ phase aligns homogeneously along $\textbf{R}$. In the mesotwist cell thickness range, the minimum transmitted light intensity increases with cell thickness and its minimum shifts away from $\gamma = 90^{\circ }$. At a critical thickness $d \approx 4.7 ~\mu \text {m}$, where the oppositely twisted domains are observed (Fig. 5d) a substantial amount of light is transmitted.

**Fig. 9:**
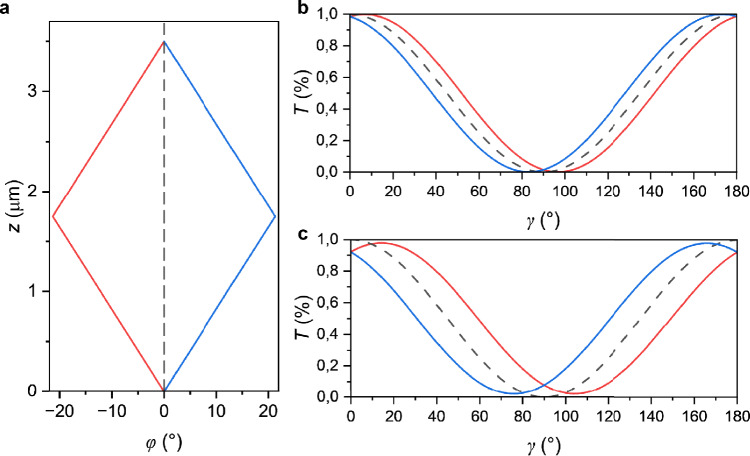
Simulated transmitted light intensity in a synpolar cell with thickness 3.5 $\mu$m for mesotwisted structures. (**a**) The simulated director structures. Here $\varphi$ represents the angle between the local director and the bottom alignment and *z* the distance from the bottom surface. In each structure the absolute value of the local twist is the same everywhere, about $12^\circ /\mu$m. The dashed black line represents a uniform configuration for comparison. (**b**) The simulated transmitted light intensity for the wavelength 500 nm. (**c**) The simulated transmitted light intensity for the wavelength 600 nm. The blue and red curves in (**b**) and (**c**) each correspond to the mesotwist structure with the same color in (a). The dashed line represents a uniform structure for reference.

To verify that the proposed mesotwist would really cause abscence of extinction and optical assymmetry upon clockwise and counter-clockwise decrossing, a simulation of the transmission was made using Jones calculus for two different wavelengths. First, an assumption was made that the local pitch would be approximately constant everywhere in the cell, but with changing twist sense along *d*. Results from one such simulation is shown in Fig. 9 for two different wavelengths in a synpolar $3.5\, \mu$m cell. In this case, the local twist was assumed to be about $12^\circ /\mu \text {m}$, with sign inversion at the midplane. Some other possible configurations can be found in the Supplementary Information. There is no evidence indicating that the specific configurations used here is the same as what appears in a real FNLC. However, the results confirm that in general mesotwist may let through light of some colors between crossed polarizers, and that it will show a non-symmetric color variation upon decrossing. This makes it a viable explanation for the observations we have made.

## Discussion

The fact that we observe twisted domains also in synpolar FNLC cells is more intriguing than the twisted domains which can be observed in antipolar cells^10^. While the latter can be readily explained by the in-plane polar surface anchoring being incompatible with a non-twisted structure, there must be another driving force for the twisted states in the synpolar case. We propose the breaking of mirror symmetry is related to the predictions of Khachaturyan^14^, Lavrentovich et al.^9^ and recently by Paik and Selinger^18^, that a ferroelectric nematic should spontaneously form a twisted structure to reduce the electrostatic energy of the sample. This is analogous to samples of solid state ferroelectrics that tends to break up in domains with opposite polarity^13^. In the ferroelectric nematic there is no crystal lattice and there is more freedom for the medium to reorient $\textbf{P}$, e.g. to form a continuous twist, where the total polarization is canceled over one full turn of the twist. But any twist deformation is related to an elastic penalty that counteracts the twist and therefore the lowest energy configuration is found to be a twisted state with a certain ”pitch”.

However, the theoretical works mentioned above address the case of a cylindrical sample with a certain radius, and the radii (or characteristic domain widths) are generally nontrivial to define in real sample cells. Furthermore, the equilibrium pitch also depends on the Debye screening length^18^. In the regime of weak screening the equilibrium pitch goes towards infinity, i.e. there should be no polarization driven twist at high ion concentrations. Paik and Selinger show that a FNLC sample can minimize its total energy by forming twisted states that cancel the total polarization, i.e. for which the total twist is $2\pi n$, where *n* is an integer number. In principle, this result is in line with our observations. In the thinnest part of the wedge, (Fig. 5a,b) and in the sandwich cell with $d=1.6 ~\mu$m (Fig. 4a-c), we find a non-twisted, i.e., uniform, state. We suggest that the elastic penalty for a finite polarization driven twist is too high at this small cell gap. In the thickest part of the wedge (Fig. 5d) and the thick sandwich cell (Fig. 4d-f) the material can, on the other hand, form a $2\pi$ twist, either right- or left-handed, to cancel the total polarization. In the intermediate thickness range, the material can make a compromise: It can start to twist one way with the equilibrium pitch, and then, twist back to the original state, again with the equilibrium pitch, but with the opposite chirality, see Fig. 7. This is possible because the right- and left-handed twisted states are degenerate. This proposed mesotwisted state cannot appear in a chiral nematic, where the right and left-handed twists are non-degenerate.

However, the mesotwist configuration is not unique to the wedge cell; a similar texture can be observed in a flat synpolar sandwich cell with a cell gap within the same range (2–4 $\upmu$m) as that in which the mesotwist is observed in the wedge cell (see Supplementary Information). In a flat synpolar cell between crossed polarizers, the mesotwist state can easily be confused with a uniform state, such that measurements of birefringence may be misinterpreted if caution is not taken.

**Fig. 10:**
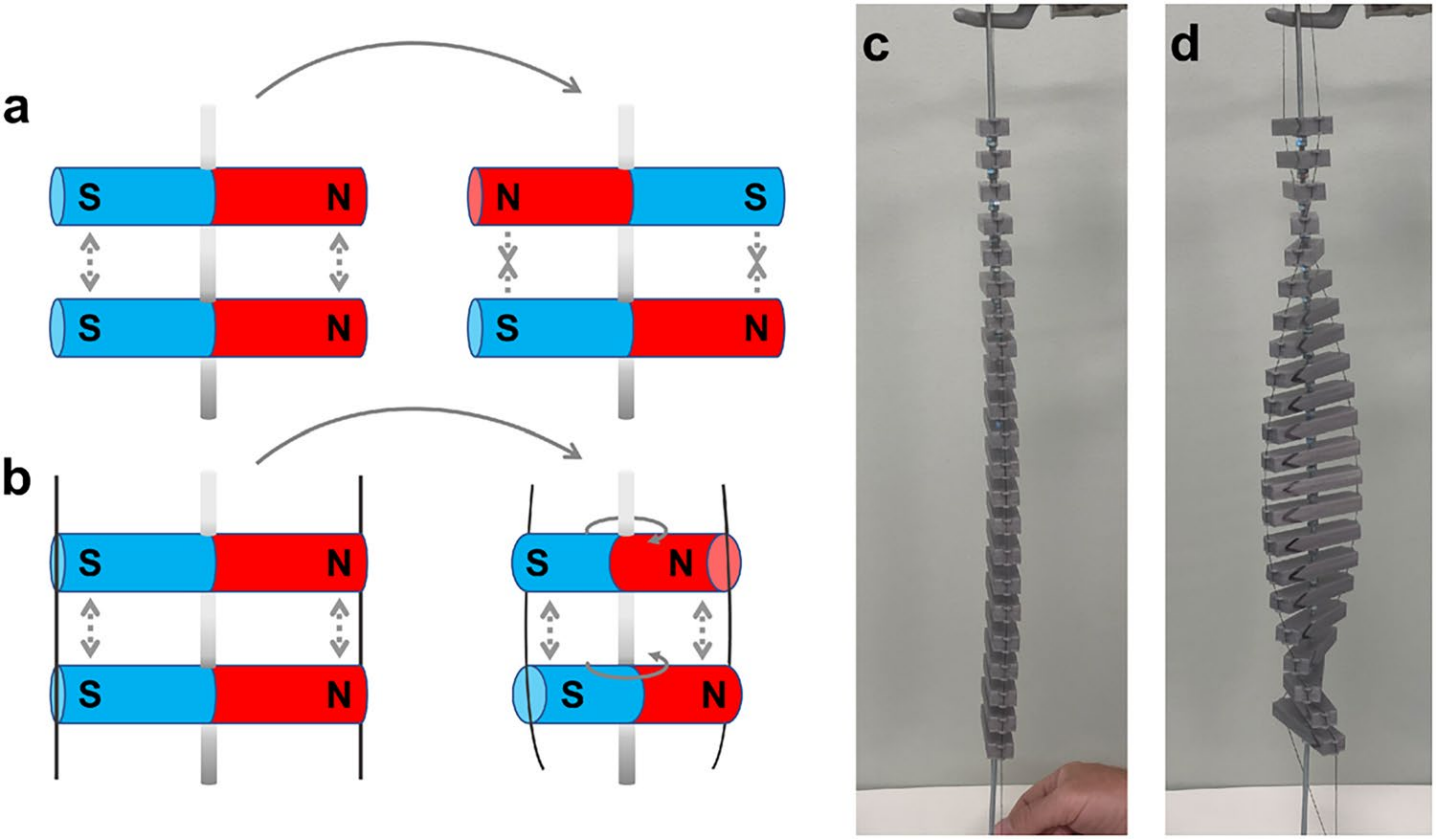
A physical analogy of the presented Gedankenexperiment. The mechanisms behind the twist are shown in (**a**) and (**b**), with and without elastic threads attached respectively. Dashed grey arrows represent attractive and repulsive magnetic forces respectively, and dark blue lines represent elastic threads. Photographs of the full model are shown in (**c**) and (**d**) with different amounts of tension in the thread. As the magnets in the model are too weak to stretch out an elastic thread at all, different amounts of tension is instead applied by pulling on the strings that hold the magnets in place.

The mesotwist can be imagined using a ferromagnetic analogy in a simple Gedankenexperiment. Picture a ferromagnetic rod, representing the polarization field of an FNLC. Two of these rods forced to be near each other side by side and parallel with the polarization in the same direction will have a high magnetic energy. If they are free to rotate but not move they will end up anti-parallel in order to minimize the magnetic energy in the system, cf. Fig. 10a. Now imagine that instead of rotating freely, they are held in place by elastic threads that represent the elasticity of the director. In this case, the magnets are not able to become anti-parallel, but they can deviate from being parallel, cf. Fig. 10b. Depending on the strength of the magnetic dipoles as well as the elasticity of the thread, some balance will be found where the total energy of the system is minimized, with some sacrifices in elastic energy and some in magnetic energy. If many of these magnets are put in a 1D lattice, and connected by an elastic thread counteracting their mutual rotation, as shown in Fig. 10c,d, a twist will appear which is similar in nature to the twist seen in an FNLC. No part of the system is chiral and the twist therefore has no preferred twist sense. This structure tends to naturally vary its twist sense along the lattice direction. Alignment layers can be simulated by holding the magnets in each end in certain orientations, in which case a mesotwist will appear. To get a full $2\pi$ twist something discontinuous must happen, namely that the magnets in one end must be allowed to turn, since a ”bulk disclination” can not be introduced in this model.

If the elastic force in the magnet structure is strong and the spontaneously appearing twist is therefore small, so that the total twist if the whole system was forced to have a constant twist sense would be less than $2\pi$, forcing a $2\pi$ twist would increase the energy in the system and require some force to keep the end magnets in place. This is analogous to an FNLC in a thin cell, where the pitch necessary to fit twist a full $2\pi$ within the cell gap is shorter than what would be energetically favorable. Interestingly, one can not increase the energy of such a system by doing the opposite and forcing a net zero twist onto a system where a lot of twist is energetically optimal. This is because the mesotwist can be arbitrarily large, and as long as it is allowed to switch twist sense at least once along the lattice direction it can reach a net zero twist across the system while keeping a constant and optimal amount of twist everywhere. This phenomenon is likely the reason why the twist domains in Figs. 5 and 6 do not appear at one clearly defined thickness, but are introduced at about $4.7\,\mu$m and increase gradually with the thickness. While there is some energetic advantages in a $2\pi$ twist at larger thicknesses, such as the phenomenon described by Paik and Selinger^18^, the difference in energy is not strong enough to create a clear vertical boundary at a critical thickness marking the end of the mesotwist regime and the beginning of the $2\pi$ twist regime.

## Methods

### Commercial liquid crystal cells

We used commercial LC cells with cell gaps of $d = 1.6~\mu \text {m}$ (purchased from AWAT PPW Company (Warsaw, Poland)) and $d = 5.0~\mu \text {m}$ (INSTEC (Boulder, Colorado, USA)). The glass cell substrates have an Indium-Tin-Oxide (ITO) electrode layer with a resitivity of 100 $\Omega$/square and an alignment layer of rubbed polyimide with 0 deg rubbing angle between the top and bottom substrates (synpolar).

### Wedge cells

To fabricate the wedge cells, 75 mm x 75 mm glass plates covered with ITO were used. The plates were cleaned using a mixture of deionised water:ammonia (concentration 25%):hydrogen peroxide (concentration 30%) in relations 6:1.2:1.2. The mixture was held in a plastic container standing in an ultrasonic bath. The glass plates were placed in the bath for four 10-minute cycles, and rinsed between each cycle. Finally, the plates were dried using a nitrogen blow dryer and then placed in an oven at $100^\circ$C for 1 h.

After cleaning, each plate was spin-coated with PI2610 (DuPont), diluted to 0.5% concentration in dimethylsulfoxide (DMSO), at 5000 rpm for 30 s, pre-baked at $100^\circ$C and cured in an oven at $300^\circ$C for 3 h. An LCTec Automation commercial substrate rubbing machine was used to apply linear rubbing to the PI layers.

Strings of UV-curing glue (Norland Adhesive 68) was dispensed on one of the substrates using a home built programmable glue dispenser. Two sets of strings were dispensed. One set contained glue mixed with $20-27 ~\mu \text {m}$ PMMA spacers and the other set had no spacers. A second substrate were put on top of the first substrate, carefully aligning the mutual rubbing directions in parallel. After assembly, the plates were pressed together using vacuum bags, according to the scheme developed in Podolskyy et al.^22^. Each assembled pair of plates was then cured with UV light for 10 minutes, and diced into 19.0 mm long wedge cells.

### Thickness measurement

The thickness of the wedge cell was calculated using an interferometry measurement taken with a UV-Vis spectrometer (model: Ocean FX, Ocean Insight) attached to an optical polarizing microscope (model: DM LM/P, Leica Microsystems GmbH) via a UV-Vis optical fiber (Ocean Insight).

### Transmitted light intensity measurements

The transmitted light intensity was collected in visible light range by the fiber optic spectrometer (details above). Measurements were made with white light and using an orange interferometric filter with $\lambda =589$ nm, placed on top of the first linear polarizer.

### Transmitted light intensity simulations

Jones calculus was used to calculate the theoretical transmitted light intensity for the same wavelengths that were used in the experimental measurements. The cell was approximated as 1000 uniform and equally thick slabs, regardless of cell thickness. A mesotwist was assumed where the local pitch was kept constant at $12^\circ /\mu \text {m}$ and the handedness switched either once at $z=d/2$ (Fig. 9a) or twice with a distance $\Delta z=d/2$ between them (Supplementary Figure 1).

## Supplementary Information

Supplementary Information.

## Data Availability

All data supporting the findings of this study are included in this article and its Supplementary Information file. Should any raw data files be needed in another format they are available from the corresponding author upon reasonable request. Simulation code is available on Github^23^: https://doi.org/10.5281/zenodo.17900623.
