# Supplementary material for: Polarization-driven twisted states in ferroelectric nematic liquid crystals under confinement
Source: Sci Rep. 2026 Apr 17;16:12710. doi: 10.1038/s41598-026-48218-7 (PMC13090344; doi:10.1038/s41598-026-48218-7)
Supplement: Supplementary file 1 — Supplementary Information. [file 41598_2026_48218_MOESM1_ESM.pdf]

Supplementary Figures: Polarization-driven twisted states in ferroelectric nematic liquid crystals under confinement

Anna Savchenko[1,+], Ebba Grönfors[2,+], Rachel Tuffin[3], Melanie Klasen-Memmer[3], Per Rudquist[2], and Frank Giesselmann[1,*]

[1]Institute of Physical Chemistry, University of Stuttgart, 70569 Stuttgart, Germany
[2]Department of Microtechnology and Nanoscience, Chalmers University of Technology, 41296 Gothenburg, Sweden
[3]Merck Electronics KGaA, 64293 Darmstadt, Germany
[*]f.giesselmann@ipc.uni-stuttgart.de
[+]these authors contributed equally to this work

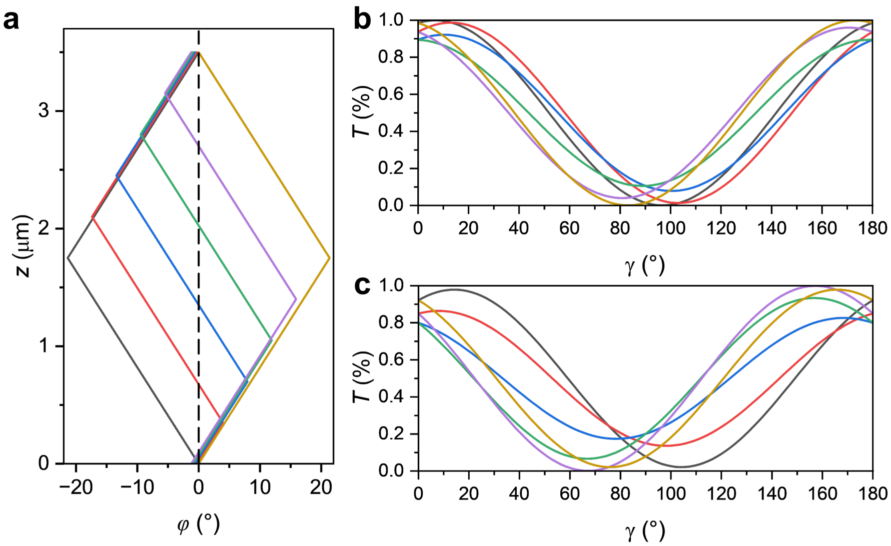

Fig. S1. Simulated transmitted light intensity in a synpolar cell with thickness 3.5 μm for different combinations of mesotwisted structures. a) The simulated director structures. Here φ represents the angle between the local director and the bottom alignment and z the distance from the bottom surface. In each structure the absolute value of the local twist is the same everywhere, about $12^\circ/\mu$m. The dashed black line shows the rubbing direction. A small offset has been added to φ for each structure to make each line visible, but in the simulations $\varphi_{z=0} = \varphi_{z=3.5} = 0$ for all structures. b) The simulated transmitted light intensity for the wavelength 500 nm. c) The simulated transmitted light intensity for the wavelength 600 nm. The curves in (b) and (c) each correspond to the mesotwist structure with the same color in (a).

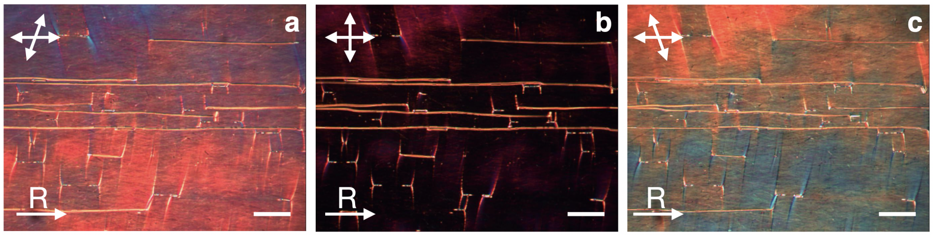

Fig. S2. AUUQU-2-N in a flat synpolar cell at 62°C. The cell gap is just below 4.0 μm. Under crossed polarizers, one can see a characteristic continuous spatial fluctuation between different colors, in this case black and red, likely caused by variations in the amount of mesotwist. The cell can never be rotated such that there is extinction of light everywhere. Upon ±20° decrossing, non-symmetric colours are seen. There are no distinct twist domains, as there would be if there was a 2π twist. The scalebar is 200 μm.

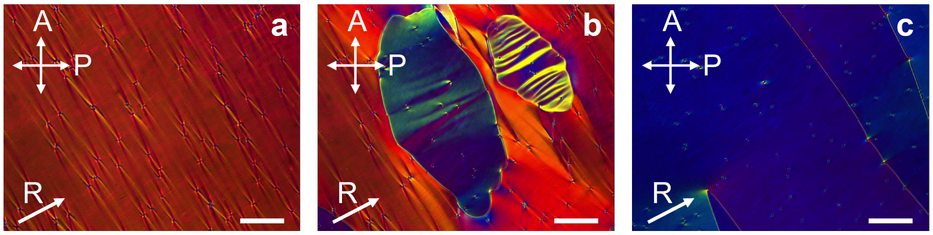

Fig. S3. Formation of domain walls in a flat 5.0 μm cell with synpolar anchoring conditions. After transition from SmZ_A to N_F, below 70°C at first we observe a uniform texture with conic defects, which are normal to rubbing direction (a). Upon cooling structural changes take place (b). At around 69°C, twist domain walls are formed also normal to **R**, while the defects now appear to be along the **R**. The scalebar is 200 μm.

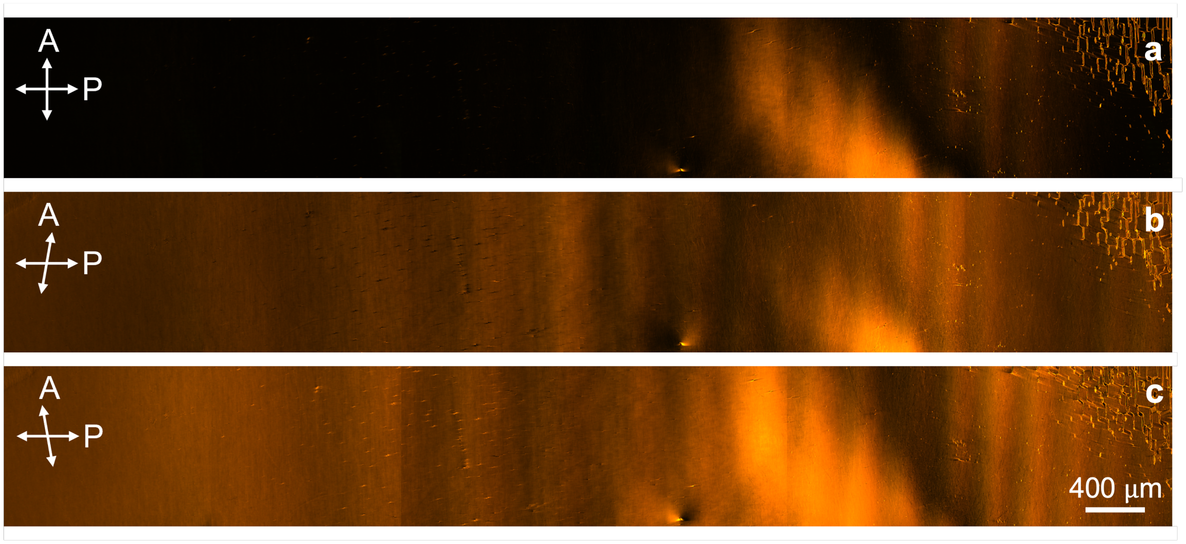

Fig. S4. Panorama picture of the thinner half of the synpolar wedge cell filled with the N_F phase under crossed (a) and ±25° decrossed polarizers (b,c) with monochromatic light. The wavelength is $\lambda = 589$ nm.

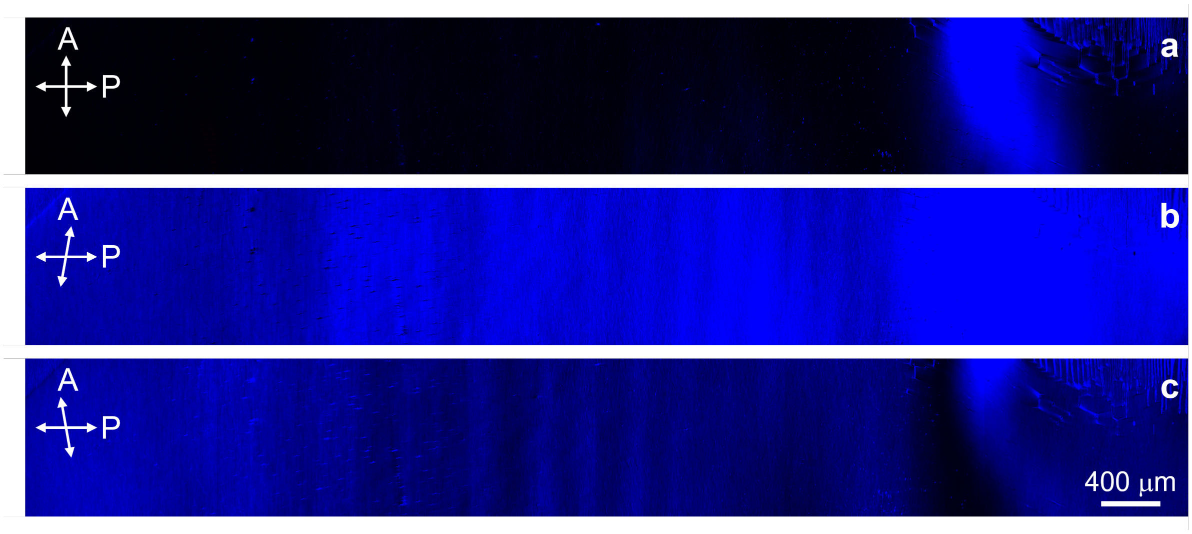

Fig. S5. Panorama picture of the thinner half of the synpolar wedge cell filled with the N_F phase under crossed (a) and $\pm 25°$ decrossed polarizers (b,c) with monochromatic light. The wavelength is $\lambda = 450$ nm.
